# Habitat dynamics, marine reserve status, and the decline and recovery of coral reef fish communities

**DOI:** 10.1002/ece3.934

**Published:** 2014-01-13

**Authors:** David H Williamson, Daniela M Ceccarelli, Richard D Evans, Geoffrey P Jones, Garry R Russ

**Affiliations:** 1 ARC Centre of Excellence for Coral Reef Studies, James Cook UniversityTownsville, Queensland, 4811, Australia; 2 School of Marine and Tropical Biology, James Cook UniversityTownsville, Queensland, 4811, Australia; 3 Department of Parks & Wildlife17 Dick Perry Ave., Kensington, Perth, Western Australia, 6151, Australia; 4 Oceans Institute, School of Plant Biology, University of Western AustraliaCrawley, Western Australia, 6009, Australia

**Keywords:** Coral reef community dynamics, disturbance, Great Barrier Reef, Marine reserves, *Plectropomus* spp

## Abstract

Severe climatic disturbance events often have major impacts on coral reef communities, generating cycles of decline and recovery, and in some extreme cases, community-level phase shifts from coral-to algal-dominated states. Benthic habitat changes directly affect reef fish communities, with low coral cover usually associated with low fish diversity and abundance. No-take marine reserves (NTRs) are widely advocated for conserving biodiversity and enhancing the sustainability of exploited fish populations. Numerous studies have documented positive ecological and socio-economic benefits of NTRs; however, the ability of NTRs to ameliorate the effects of acute disturbances on coral reefs has seldom been investigated. Here, we test these factors by tracking the dynamics of benthic and fish communities, including the important fishery species, coral trout (*Plectropomus* spp.), over 8 years in both NTRs and fished areas in the Keppel Island group, Great Barrier Reef, Australia. Two major disturbances impacted the reefs during the monitoring period, a coral bleaching event in 2006 and a freshwater flood plume in 2011. Both disturbances generated significant declines in coral cover and habitat complexity, with subsequent declines in fish abundance and diversity, and pronounced shifts in fish assemblage structure. Coral trout density also declined in response to the loss of live coral, however, the approximately 2:1 density ratio between NTRs and fished zones was maintained over time. The only post-disturbance refuges for coral trout spawning stocks were within the NTRs that escaped the worst effects of the disturbances. Although NTRs had little discernible effect on the temporal dynamics of benthic or fish communities, it was evident that the post-disturbance refuges for coral trout spawning stocks within some NTRs may be critically important to regional-scale population persistence and recovery.

## Introduction

Cycles of disturbance and recovery are a key feature of coral reef ecosystems, and occasional acute disturbances are considered integral to maintaining high species diversity (Connell 1978; Rogers 1993). However, if the intensity and frequency of disturbance exceeds certain thresholds, communities may not be able to fully recover between disturbance events and the health of the coral reef communities will gradually decline (Aronson et al. 2005; Thompson and Dolman 2010). In some cases, this has led to reefs undergoing a “phase shift” to a stable algal-dominated state (Bellwood et al. 2004). Cycles of habitat change and long-term habitat degradation have major flow-on effects on the structure of reef fish communities (Jones and Syms 1998; Jones et al. 2004; Graham et al. 2006; Wilson et al. 2008). One of the most commonly proposed direct management tools expected to buffer coral reef communities against disturbance-driven declines are no-take marine reserves (NTRs) (Graham et al. 2011). Networks of NTRs have been shown to be an effective tool for the protection of exploited species, often leading to increased density, size, age, and per-capita fecundity of these species inside reserves (Halpern 2003; Lester et al. 2009; Molloy et al. 2009), and subsequent adult spill-over (Abesamis et al. 2006; Goni et al. 2010) and recruitment subsidy benefits (Harrison et al. 2012) outside reserves. However, the ability of NTRs to protect coral communities, habitat quality, reef fish biodiversity, and key fishery species in the face of major environmental impacts, such as storms, floods, or coral bleaching events, is less understood.

It has been hypothesized that effective NTR networks can promote healthy and productive coral reef ecosystems that have a greater capacity for limiting declines and enhancing recovery from disturbance events (Almany et al. 2009; Graham et al. 2011). The empirical evidence for such effects has been contradictory (Jones et al. 2004; Claudet et al. 2011). A study by Jones et al. (2004) found that coral reef fish biodiversity declined at the same rate in both reserves and fished areas in response to declines in coral cover. Other studies have demonstrated enhanced recovery from disturbance within NTRs (McClanahan et al. 2007; Babcock et al. 2010; Mumby and Harborne 2011). The conflicting results provide no clear picture of the relative roles of reserves and other factors in influencing the magnitude and rates of decline and recovery of coral reefs. To date, too few studies have examined habitat and fish dynamics in both NTRs and fished areas over full cycles of disturbance and recovery.

The response of coral reef benthic communities to disturbance and the subsequent recovery trajectories depends not only on the type, frequency, and severity of disturbances, but also on the composition of coral assemblages and their pre-disturbance condition. For instance, branching and plating acroporid corals are relatively vulnerable to damage, but they also tend to be fast growing and quick to recover (Carpenter et al. 2008). Furthermore, local acclimation and/or adaptation within genera and species may be critically important in determining the degree to which coral reef communities are impacted by disturbances. For example, corals living on near-shore reefs may be more resistant to sedimentation and exposure to low-salinity water than those accustomed to the conditions on offshore reefs (Flores et al. 2012).

The response of reef fishes to habitat change also varies depending on the ecology and life history of the species. Coral feeders and small habitat specialists are generally much more vulnerable to declining coral cover, or loss of certain types of corals, than generalist species (Munday 2004; Berumen and Pratchett 2008). Species of fish with larger bodies are more likely to fluctuate in response to changes in prey abundance or the structural complexity of the benthos, rather than simply the abundance of live coral (Wilson et al. 2006, 2009). However, in areas with low underlying rugosity of the coral reef matrix, corals provide structure at a scale that is relevant for most fish species (MacNeil et al. 2009). Overall, reductions in fish species diversity in response to habitat loss may have little functional consequence in highly diverse systems such as coral reefs, where many species can perform the same ecological role (Bellwood and Hughes 2001; Bellwood et al. 2002; Floeter et al. 2004). Therefore, assessing reef fish community responses to disturbance at the level of functional groups may provide greater insight into the magnitude and consequences of the impact than assessing species-specific changes.

Although it has been shown that populations of targeted reef fish and invertebrate species can build rapidly within adequately protected NTRs (Russ et al. 2008; Babcock et al. 2010), population gains can be slower in some systems and continue to accrue over decadal time scales (Russ and Alcala 2010). It has also been shown that effective NTR networks can enhance the persistence of populations of targeted reef fishes, such as coral trout (*Plectropomus* spp.) and tropical snappers (Lutjanidae), by protecting spawning stock biomass and providing important sources of juvenile recruitment to both reserves and fished areas (Harrison et al. 2012; Almany et al. 2013). However, disturbance events often impact communities in both NTRs and fished areas, and the degree to which NTRs may maintain high densities and biomass of exploited fishes following severe disturbance to the benthos is unknown. Reserves may play a critical role in population and community recovery following disturbances, but only if they can provide effective refuges in times of disturbance.

The overall aim of this study was to quantify temporal cycles of benthic habitat decline and recovery, assess its impact on the dynamics and structure of coral reef fish communities, and assess the role of NTRs in minimizing degradation or promoting recovery of coral reef fish communities. The study focused on the Keppel Island Group, southern Great Barrier Reef (GBR), where coral reefs were subjected to two major acute disturbance events during a long-term monitoring program: a severe coral bleaching event in 2006 and a freshwater flood plume from the nearby coastal river system, the Fitzroy River, in 2011. The 2006 coral bleaching event was relatively localised and predominantly impacted reefs in the Keppel Islands and the southern GBR. Previous mass bleaching events in 1998 and 2002 impacted reefs over broader areas of the GBR, but had little impact in the Keppel Islands (Berkelmans et al. 2004; Diaz-Pulido et al. 2009). While relatively minor flood plumes frequently affect inshore GBR reefs, the flood plumes of 2010/2011 were particularly severe and prolonged (Brodie et al. 2012). In the Keppel Islands, fast-growing branching acroporid corals grow on relatively low relief fringing reef slopes and flats (Diaz-Pulido et al. 2009) and provide much of the reefs' structural complexity. A network of NTRs covers approximately 28% of the fringing coral reefs in the Keppel Islands. Monitoring programs conducted within the Great Barrier Reef Marine Park (GBRMP) have detected significant increases in the density and biomass of key fishery targeted species such as coral trout (*Plectropomus* spp.) within NTRs (Evans and Russ 2004; Williamson et al. 2004; Russ et al. 2008). However, the role of NTRs in protecting exploited species in habitats that are highly disturbed by bleaching and flooding has not been examined, nor has the role of NTRs in promoting recovery following such disturbance events.

The following specific questions were addressed: (1) What were the patterns in the decline and recovery of benthic communities in response to the disturbances and did NTRs mitigate against the impacts or promote recovery? (2) What were the patterns of change in reef fish community structure in response to changes in the benthic habitat and did NTRs ameliorate the effects of the disturbances on fish? (3) Did NTRs buffer populations of important fishery target species such as coral trout (*Plectropomus* spp) against the effects of disturbance-driven loss of benthic habitat? (4) Can NTRs provide post-disturbance refuges of spawning stocks of fishery target species, which aid local recovery through recruitment and boost population persistence?

## Materials and Methods

### Study area, history of disturbance, and marine park protection

**Figure 1:**
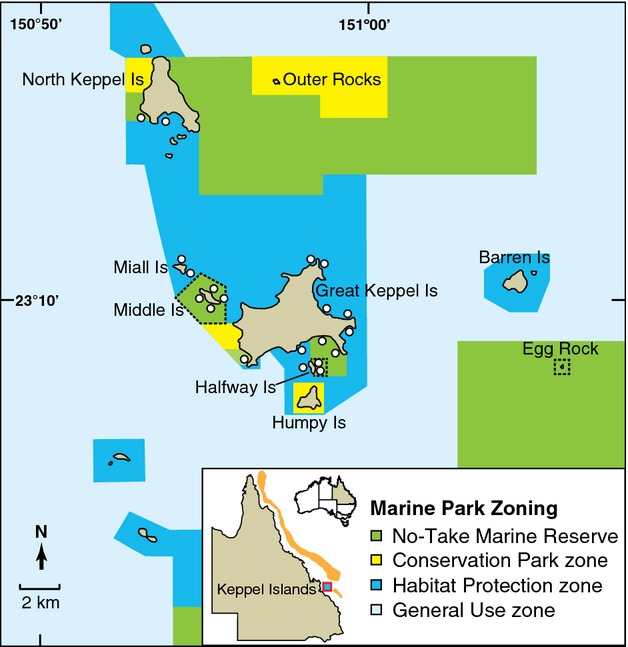
Map of the Keppel Island group showing the approximate location of 20 monitoring sites (white markers) and the arrangement of management zones. No-take marine reserves (NTR – Marine National Park zones), shaded green, are divided into “old NTRs” (established in 1987) and “new NTRs” (established in 2004). Black dashed lines indicate previous old NTR boundaries. All other zones are open to fishing. Conservation Park zones (shaded yellow) exclude commercial fishing but permit limited recreational hook-and-line fishing. Habitat Protection zones (dark blue) exclude demersal trawling but permit hook-and-line and spear fishing. General-use zones (light blue) allow all types of fishing.

This study was conducted in the Keppel Island group (23°100′S, 150°570′E) within the southern section of the Great Barrier Reef Marine Park (GBRMP), Australia (Fig. 1). Multiple-use management zoning plans were first implemented within the GBRMP in 1987, and from that time until 2004, approximately 5% of the marine park area was protected within a network of NTRs. The GBRMP was rezoned in July 2004, and the area protected within NTRs was increased to cover approximately 33% of the total area (and 33% of the coral reefs). The principal objective of the new zoning plan was to increase biodiversity protection and ecosystem resilience by allocating a proportion of the area within each of seventy identified bio-regions into an interconnected network of NTRs (Fernandes et al. 2005). At the Keppel Islands, fringing coral reefs cover approximately 700 hectares, of which 196 hectares (˜28%) is protected within a network of NTRs. Three reef areas have been protected within NTRs since 1987, while four additional reef areas were designated as NTRs in July 2004.

Two distinct climatic disturbance events impacted fringing reefs in the Keppel Islands during the monitoring period (2004–2011). In March 2006, a sustained period of elevated sea temperature triggered a severe coral bleaching event. Five years later, several intense rainfall events between December 2010 and March 2011 produced a major flood of the Fitzroy river catchment and resulted in a freshwater flood plume that engulfed the Keppel Islands for several weeks. The 2006 bleaching event impacted all reef habitats (flat, crest, slope) in most monitoring sites. The 2011 flood plume event tended to have the largest impact on reef flats, crests, and shallower sections of reef slopes to a depth of approximately 2 m below low water datum. However, most fringing reefs in the Keppel Islands reach a maximum depth of less than 12 m and some only reach 4–5 m. The maximum tidal range in this region is approximately 5 m; thus, the flood plume inundated most, or the entire reef slope habitat at the majority of the monitoring sites.

### The monitoring program

Reef fish and benthic communities were surveyed at twenty sites in the Keppel Island group on five occasions between 2004 and 2011 using underwater visual census (UVC). Six of the monitoring sites were located within “old” (1987) NTRs, four sites were within “new” (2004) NTRs, and ten sites were located in areas that have remained open to fishing (Fig. 1). Baseline UVC surveys were conducted at all sites on a single occasion prior to the implementation of the new NTRs in July 2004. Post-NTR implementation surveys were carried out in 2006, 2007, 2009, and 2011.

Approximately 220 species of diurnal, noncryptic reef fish, in 17 families (see Table S1, supplementary material) were surveyed on five replicate transects within each site. Transects were deployed on reef slopes, parallel to the reef crest, and within a depth range of 3–9 m, depending on the structure of the reef slope at each site. Transects were 50 × 6 m (300 m^2^ survey area) for all species other than pomacentrids and small labrids, which were surveyed during return transect swims using a transect width of 2 m (100 m^2^ survey area). All fish surveys were conducted on SCUBA by two fish observers who swam in close proximity to each other. One observer surveyed the predatory species (predominantly Lethrinidae, Lutjanidae, Serranidae, Haemulidae, and larger species of Labridae), while the other surveyed the roving herbivores (predominantly Acanthuridae, Scaridae, and Siganidae) and other nonfishery target groups (Chaetodontidae, Pomacanthidae, Pomacentridae, and small species of Labridae). A third diver swam approximately 5 m behind the fish observers deploying the transect tapes. This synchronous transect deployment technique minimized diver avoidance or attraction behaviors of certain fishes and improved the accuracy of the UVC. All larger fish species were assigned into 5-cm-length classes. Fish observers conducted length estimation calibration using fish models at the start of each survey trip. Three observers conducted all fish surveys throughout the monitoring period (DHW, RDE, and DMC).

The benthic community was surveyed using a standard line intercept survey method. Benthic point samples were recorded for every 1 m graduation along each transect (50 points per transect). Hard corals were classified as live or dead and assigned into morphological categories (branching, digitate, plate, massive, foliose, encrusting, solitary). Other categories of benthos included live soft coral, sponges, clams (*Tridacna* spp.), other invertebrates (such as ascidians and anemones), macro-algae, coral reef pavement, rock, rubble, and sand. Reef structural complexity was estimated using a five-point scale for both reef slope angle and rugosity (Table 1). Five independent structural complexity estimates were made for each transect. Visibility was recorded on each transect and typically ranged from 6 to 12 m. Surveys did not proceed if visibility was less than 5 m.

**Table 1:** Description of categories for reef slope and rugosity, estimated visually on each transect in the Keppel Island group.

Category	Description of reef slope and rugosity
1	Reef slope 0–10°. Expanses of rubble and sand with some small scattered bommies
2	Reef slope <45°. Bommies dispersed among mostly rubble and sand
3	Reef slope ˜45°. Small rubble and sand patches among bommies and/or coral structure
4	Reef slope >45°. Complex coral structure, bommies, some small over-hangs, holes, and caves
5	Reef slope ˜90°. High reef complexity, large over-hangs, holes, caves, and bommies

### Statistical analysis

Raw benthic community data were converted to percent cover estimates. Fish density estimates were expressed as individuals 1000 m^−2^, obtained by multiplying raw counts by 3.33 for fish species surveyed on 50 × 6 m (300 m^2^) transects, and by 10 for fishes surveyed on 50 × 2 m (100 m^2^) transects. All fish species were classified into trophic functional groups, and analyses were conducted using these groupings (see Table S1, supplementary material). The significance of variation in live hard coral cover, habitat complexity (rugosity index), the density of fish functional groups, and the density of coral trout (*Plectropomus* spp.) as an independent group, were tested between years and zones using repeated measures analysis of variance (ANOVA). Year (2004–2011) was treated as the repeated measure, while zone (new NTR, old NTR and fished) and site (nested within zone) were treated as fixed factors, with transects as replicates. Repeated measures ANOVA was also used to test for differences in the mean length of *Plectropomus* spp. between years and zones, by using the mean length values for each site (site means) as the replicates. All data were subjected to Shapiro–Wilk's normality test, Cochran's C homogeneity of variance test, and Mauchly's sphericity test. Transformations, √(x + 1) or log_n_(x + 1), of the raw data were conducted if required to conform to ANOVA assumptions.

We used nonmetric multidimensional scaling analysis (MDS, Clarke and Gorley 2006) on the Bray–Curtis resemblance matrix of transformed log_n_ (x + 1) cover of benthic categories and square-root density of each fish functional group to partition management zone (new NTR, old NTR, and fished) characteristics in different years. We then conducted a nonmetric, one-way, pairwise analysis of similarity (ANOSIM) among the groups, and a SIMPER analysis (Clarke and Warwick 2001) to determine the species or groups that most strongly accounted for the similarities and differences between zones and years. To determine the relative contribution of the original variables (i.e., benthic categories or fish functional group) to the final MDS solution, each variable was projected onto the ordination space. Vectors were calculated using the partial regression coefficients of the original variables within the two dimensions of the MDS, and the lengths of the vectors were set proportional to the squared multiple correlation coefficient.

A generalized linear mixed-effects model was used to test the degree of influence of live hard coral (LHC) cover on the density of fish functional groups and the *Plectropomus* spp. group. Year and LHC were treated as fixed factors, while site was treated as a random factor and transects were used as replicates.

To examine the distribution of post-disturbance refuges for the key fishery targeted species on these reefs (*Plectropomus* spp.), the mean density across all sites (both NTR and fished) and survey years was calculated and this was set as the threshold density. Mean coral trout densities were calculated for all sites and partitioned within fished zones, old NTRs (1987), and new NTRs (2004) for the healthy reef condition (pre-disturbance) periods of 2004 and 2009 and for the degraded (post-disturbance) periods of 2006 and 2011. Sites that supported coral trout densities above the threshold were classified as refuges.

## Results

### Temporal dynamics of the benthic community: coral cover, habitat complexity, and composition

**Figure 2:**
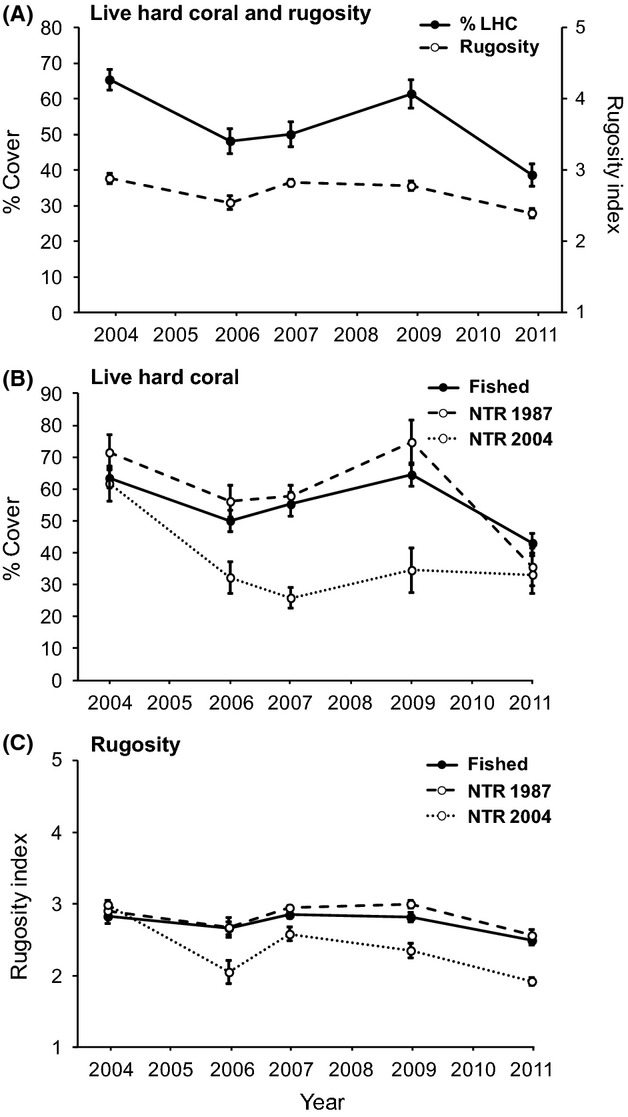
Temporal dynamics in live hard coral cover and habitat complexity (rugosity index) in the Keppel Island group between 2004 and 2011. (A) Mean live hard coral cover (LHC) and rugosity within all management zones combined (all sites pooled). (B) Mean LHC in fished zones, old NTRs (NTR 1987), and new NTRs (NTR 2004). (C) Mean rugosity in fished zones, old NTRs, and new NTRs. Error bars are ±1 SE of the mean.

Across all twenty NTR and fished sites, the overall mean cover of live hard coral declined significantly by 26% in response to the 2006 bleaching event, and by 37% following the 2011 flood plume (Fig. 2A, Table 2). Rapid recovery of hard coral cover was recorded in the period between the two disturbance events, with the mean live hard coral cover across all sites increasing by 27% from 2006 to 2009. In 2009, the overall mean cover of live hard coral was approximately 61%, and in the majority of sites, it had effectively recovered to the pre-disturbance state of 2004. Reef habitat complexity (rugosity index) also varied significantly between years, with a trajectory that mirrored the changes in hard coral cover (Fig. 2A, Table 2).

**Table 2:** Results of repeated measures analysis of variance on temporal changes in primary benthic attributes (% live coral cover and rugosity), and major fish groups, within and between management zones of the Keppel Island group.

Dependent variable	Factor_(df)_	*F*	*P*	Dependent variable	Factor_(df)_	*F*	*P*
% Live hard coral cover	Year_(4,320)_	58.2	<0.001	Corallivores	Year_(4,320)_	43.7	<0.001
	Zone_(2,80)_	28.9	<0.001		Zone_(2,80)_	61.7	<0.001
	Site (Zone)_(17,80)_	9.6	<0.001		Site (Zone)_(17,80)_	12.7	<0.001
	Year × Zone_(8,320)_	9.7	<0.001		Year × Zone_(8,320)_	4.9	<0.001
	Year × Site_(68,320)_	4.13	<0.001		Year × Site_(68,320)_	5.8	<0.001
Rugosity Index	Year_(4,320)_	70.7	<0.001	Omnivorous pomacentrids	Year_(4,320)_	18.0	<0.001
	Zone_(2,80)_	47.1	<0.001		Zone_(2,80)_	4.7	<0.05
	Site (Zone)_(17,80)_	19.6	<0.001		Site (Zone)_(17,80)_	5.8	<0.001
	Year × Zone_(8,320)_	11.7	<0.001		Year × Zone_(8,320)_	3.8	<0.001
	Year × Site_(68,320)_	7.6	<0.001		Year × Site_(68,320)_	3.5	<0.001
Fish species richness	Year_(4,320)_	40.9	<0.001	Planktivorous pomacentrids	Year_(4,320)_	19.9	<0.001
	Zone_(2,80)_	14.5	<0.001		Zone_(2,80)_	0.7	0.50
	Site (Zone)_(17,80)_	3.8	<0.001		Site (Zone)_(17,80)_	9.9	<0.001
	Year × Zone_(8,320)_	2.9	<0.01		Year × Zone_(8,320)_	3.1	<0.01
	Year × Site_(68,320)_	2.1	<0.001		Year × Site_(68,320)_	3.7	<0.001
Total Fish density	Year_(4,320)_	35.5	<0.001	Territorial pomacentrids	Year_(4,320)_	21.8	<0.001
	Zone_(2,80)_	0.4	0.650		Zone_(2,80)_	48.2	<0.001
	Site (Zone)_(17,80)_	10.2	<0.001		Site (Zone)_(17,80)_	8.9	<0.001
	Year × Zone_(8,320)_	3.0	<0.01		Year × Zone_(8,320)_	7.0	<0.001
	Year × Site_(68,320)_	5.0	<0.001		Year × Site_(68,320)_	7.9	<0.001
Benthic carnivores	Year_(4,320)_	39.9	<0.001	Intermediate predators	Year_(4,320)_	2.3	0.06
	Zone_(2,80)_	4.2	<0.05		Zone_(2,80)_	1.2	0.29
	Site (Zone)_(17,80)_	6.3	<0.001		Site (Zone)_(17,80)_	2.7	<0.05
	Year × Zone_(8,320)_	6.3	<0.001		Year × Zone_(8,320)_	0.2	0.99
	Year × Site_(68,320)_	6.6	<0.001		Year × Site_(68,320)_	0.5	0.99
Detritivores	Year_(4,320)_	25.2	<0.001	Large Predators	Year_(4,320)_	18.3	<0.001
	Zone_(2,80)_	15.6	<0.001		Zone_(2,80)_	65.9	<0.001
	Site (Zone)_(17,80)_	7.9	<0.001		Site (Zone)_(17,80)_	18.7	<0.001
	Year × Zone_(8,320)_	4.0	<0.001		Year × Zone_(8,320)_	1.6	0.12
	Year × Site_(68,320)_	5.4	<0.001		Year × Site_(68,320)_	1.8	<0.001
Grazers	Year_(4,320)_	29.5	<0.001	*Plectropomus* spp.	Year_(4,320)_	19.5	<0.001
	Zone_(2,80)_	4.2	<0.05		Zone_(2,80)_	75.1	<0.001
	Site (Zone)_(17,80)_	3.8	<0.001		Site (Zone)_(17,80)_	21.2	<0.001
	Year × Zone_(8,320)_	3.5	<0.001		Year × Zone_(8,320)_	2.1	<0.05
	Year × Site_(68,320)_	3.5	<0.001		Year × Site_(68,320)_	1.9	<0.001

Trajectories of decline and recovery of live hard coral were significantly different between management zones (Fig. 2B, Table 2). In fished zones and in the old NTRs, mean hard coral cover declined by approximately 21% following the 2006 bleaching event, recovered well between 2006 and 2009, and then declined significantly again (by 33% and 52%, respectively) following the 2011 flood plume (Tukey's HSD, fished zones: *P *=* *0.02; old NTRs: *P *=* *0.0002). In the new NTRs, the 2006 bleaching event led to a 48% decline in live hard coral cover. Recovery was negligible in the new NTRs between 2006 and 2009, with mean cover remaining at around 30%. The 2011 flood plume did not generate further declines in live hard coral cover within the new NTRs (Fig. 2B). During the pre-disturbance period of 2004, 80% of sites within fished zones, 100% of sites within old NTRs, and 75% of sites within new NTRs were dominated by live hard coral with at least 50% cover. During the post-disturbance period of 2011, only 30% of fished zone sites, none of the old NTR sites, and 25% of new NTR sites had retained above 50% cover of live hard coral.

Changes in habitat complexity over the monitoring period did not differ significantly between fished zones and old NTRs; however in new NTRs, complexity declined significantly in 2006 and failed to recover during the remainder of the monitoring period (Fig. 2C, Table 2).

**Figure 3:**
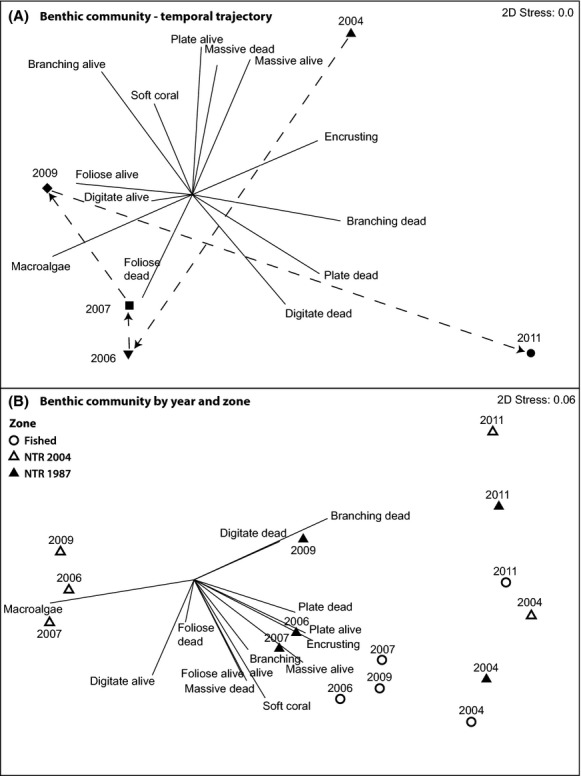
Nonmetric Multidimensional Scaling (nMDS) biplot on the Bray–Curtis similarity matrix of log (x + 1) transformed benthic community data. (A) nMDS run on annual means for all sites pooled, with temporal trajectory indicated (dashed line). (B) Annual means partitioned by fished zones, old NTRs (NTR 1987), and new NTRs (NTR 2004).

The composition of the benthic community also changed dramatically throughout the monitoring period. There was a clear shift from a community dominated by live branching, plate (predominantly *Acropora* spp.), and massive corals (mostly Poritidae and Faviidae) in 2004 to a dominance of dead foliose corals and macroalgae after the bleaching event in 2006 (ANOSIM *R* = 0.346, *P *=* *0.001). Benthic community composition changed between 2006 and 2009 toward higher proportions of live corals of varying morphologies, but not significantly so (ANOSIM *R* = 0.106, *P *=* *0.057). The flood plume disturbance of 2011 ultimately led to a dominance of dead hard corals in 2011 (ANOSIM *R* = 0.308, *P *=* *0.001, Fig. 3A). The composition of the impacted coral community in 2006 was different from the impacted community in 2011 (ANOSIM *R* = 0.450, *P *=* *0.001), with macroalgae, and live and dead branching corals being the key benthic categories separating the two post-disturbance communities (Table 3). Pre-disturbance (2004) and post-recovery (2009) benthic communities were also different (ANOSIM *R* = 0.207, *P *=* *0.001), with macroalgae and dead branching coral driving the dissimilarity (Table 3).

**Table 3:** Results of SIMPER analysis on the percent cover of benthic categories, testing dissimilarity between years and zones. Only benthic categories that accounted for at least 10% of the dissimilarity are included.

Benthic category	Contribution%	Cumulative%	Benthic category	Contribution%	Cumulative%
Groups 2004 & 2006 – Average dissimilarity = 39.2			Groups Fished & NTR 1987 – Average dissimilarity = 31.45		
Macroalgae	21.17	21.17	Soft coral	11.89	11.89
Plate alive	11.35	32.52	Macroalgae	11.33	23.21
			Encrusting	10.70	33.92
Groups 2006 & 2009 – Average dissimilarity = 35.41			Branching dead	10.58	44.49
Macroalgae	15.70	15.70	Plate dead	10.18	54.68
Plate alive	12.16	27.87	Plate alive	10.06	64.74
Digitate alive	11.37	39.24			
Branching dead	10.99	50.23	Groups Fished & NTR 2004 – Average dissimilarity = 38.77					
			Macroalgae	14.83	14.83
Groups 2009 & 2011-Average dissimilarity = 39.29			Branching dead	11.45	26.28
Branching dead	18.69	18.69	Branching alive	10.90	37.18
Macroalgae	18.21	36.91	Soft coral	10.70	47.88
Groups 2004 & 2009-Average dissimilarity = 36.60			Groups NTR 1987 & NTR 2004 – Average dissimilarity = 34.71					
Macroalgae	17.98	17.98	Macroalgae	15.59	15.59
Branching dead	13.28	31.26	Branching dead	13.44	29.03
			Branching alive	13.36	42.39
Groups 2006 & 2011 – Average dissimilarity = 41.20			Plate alive	11.10	53.48
Macroalgae	22.07	22.07	Plate dead	10.31	63.79
Branching dead	12.04	34.11	Digitate alive	10.12	73.91

Dead branching coral and macroalgae largely accounted for the differences in temporal variation in the benthic community among management zones. All three zones were dominated by a diversity of live coral forms in 2004 and by dead branching corals in 2011 (Fig. 3B). However, after the initial disturbance in 2006, macroalgae was dominant only in new NTRs, and this dominance persisted until 2009. Within fished zones and old NTRs, the shift in the benthic community attributable to the 2006 bleaching event was less dramatic than in the new NTRs. As with live coral cover, coral community structure quickly returned to a state that was closer to the original (2004) community (Fig. 3B). Temporal changes in the cover of hard corals (branching, plate, and digitate), soft coral, and macroalgae were identified as key drivers of the dissimilarity between NTRs and fished zones, and between old and new NTRs (Table 3).

### Temporal dynamics of the fish community: abundance and community structure

**Figure 4:**
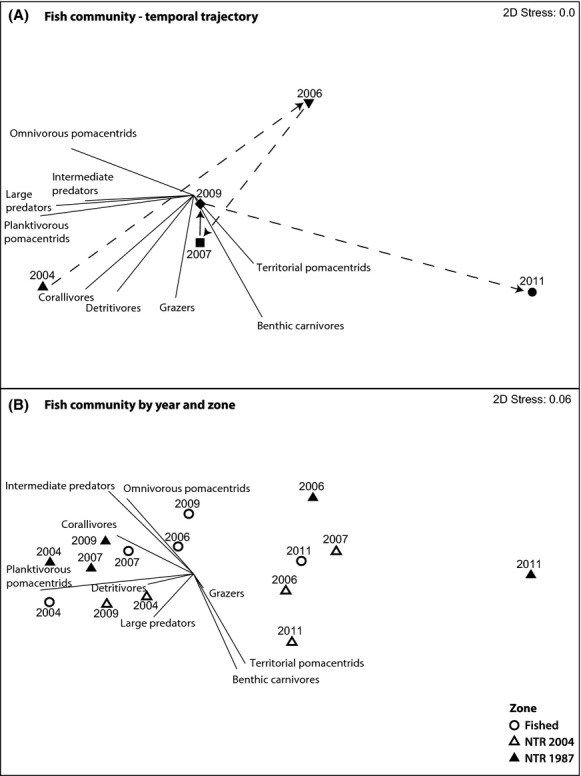
Nonmetric Multidimensional Scaling (nMDS) biplot on the Bray–Curtis similarity matrix of square-root transformed fish community data. (A) nMDS run on annual means for all sites pooled, with temporal trajectory indicated (dashed line). (B) Annual means partitioned by fished zones, old NTRs (NTR 1987), and new NTRs (NTR 2004).

Fish community structure and composition shifted significantly between 2004 and 2011 (ANOSIM *R* = 0.253, *P *=* *0.004), from an assemblage that was dominated by planktivorous and omnivorous pomacentrids, and predators, to one with a reduced abundance of most functional groups and species except benthic carnivores (particularly small labrids) and territorial (herbivorous) pomacentrids (Fig. 4A). Although fish community structure shifted considerably following both the 2006 and 2011 disturbances, the relative scale of change was greatest between the healthy reef state period of 2009 and the degraded state of 2011. The initial decline (2006), recovery (2006–2009), and subsequent decline (2011) of planktivorous pomacentrids largely defined the temporal trajectory of the fish community, with this group consistently accounting for at least 64% of the dissimilarity among years (Table 4).

**Table 4:** Results of SIMPER analysis on the mean density of fish functional groups, testing dissimilarity between years and zones. Only groups that accounted for at least 5% of the dissimilarity are included.

Fish functional group	Contribution%	Cumulative%	Fish functional group	Contribution%	Cumulative%
Groups 2004 & 2006 – Average dissimilarity = 26.76			Groups 2006 & 2011 – Average dissimilarity = 26.37					
Plank. pomacentrids	70.80	70.80	Plank. pomacentrids	64.21	64.21
Omni. pomacentrids	5.71	76.50	Terri. pomacentrids	8.11	72.32
Terri. pomacentrids	5.04	81.55	Omni. pomacentrids	7.45	79.77
			Grazers	5.28	85.05
Groups 2006 & 2009 – Average dissimilarity = 24.73			Benthic carnivores	5.12	90.16
Plank. pomacentrids	70.36	70.36			
Omni. pomacentrids	7.18	77.54	Groups Fished & NTR 1987 – Average dissimilarity = 22.72					
Terri. pomacentrids	5.21	82.75	Plank. pomacentrids	65.49	65.49
			Omni. pomacentrids	7.80	73.29
Groups 2009 & 2011 – Average dissimilarity = 26.28			Terri. pomacentrids	5.87	79.17
Plank. pomacentrids	65.37	65.37	Grazers	5.32	84.49
Omni. pomacentrids	8.69	74.06			
Terri. pomacentrids	5.86	79.92	Groups Fished & NTR 2004 – Average dissimilarity = 23.20					
			Plank. pomacentrids	61.11	61.11
Groups 2004 & 2009 – Average dissimilarity = 21.73			Terri. pomacentrids	9.57	70.68
Plank. pomacentrids	65.43	65.43	Omni. pomacentrids	8.27	78.96
Omni. pomacentrids	8.66	74.08			
Grazers	5.86	79.94	Groups NTR 1987 & NTR 2004 – Average dissimilarity = 23.16					
			Plank. pomacentrids	60.15	60.15
			Terri. pomacentrids	10.06	70.22
			Omni. pomacentrids	7.83	78.04
			Grazers	5.03	83.07

As with the benthic community, temporal trajectories in fish community structure varied between fished zones, new NTRs, and old NTRs, but not significantly so (ANOSIM *R* = 0.04, *P *=* *0.82, Fig. 4B). Prior to reserve implementation in 2004, the new NTR sites had a lower representation of planktivorous pomacentrids, obligate corallivores, and predators than fished zones and old NTRs. The shift toward benthic carnivores and territorial pomacentrids in 2006 and 2011 was most pronounced in the new NTRs. The largest shifts in overall fish community structure occurred in old NTRs between 2004 and 2006 in response to the bleaching disturbance, between 2006 and 2009 during the recovery phase, and between 2009 and 2011 in response to the flood plume disturbance (Fig. 4B). Planktivorous, omnivorous, and territorial pomacentrids consistently accounted for greater than 78% of the total dissimilarity among the three management zones (Table 4).

**Figure 5:**
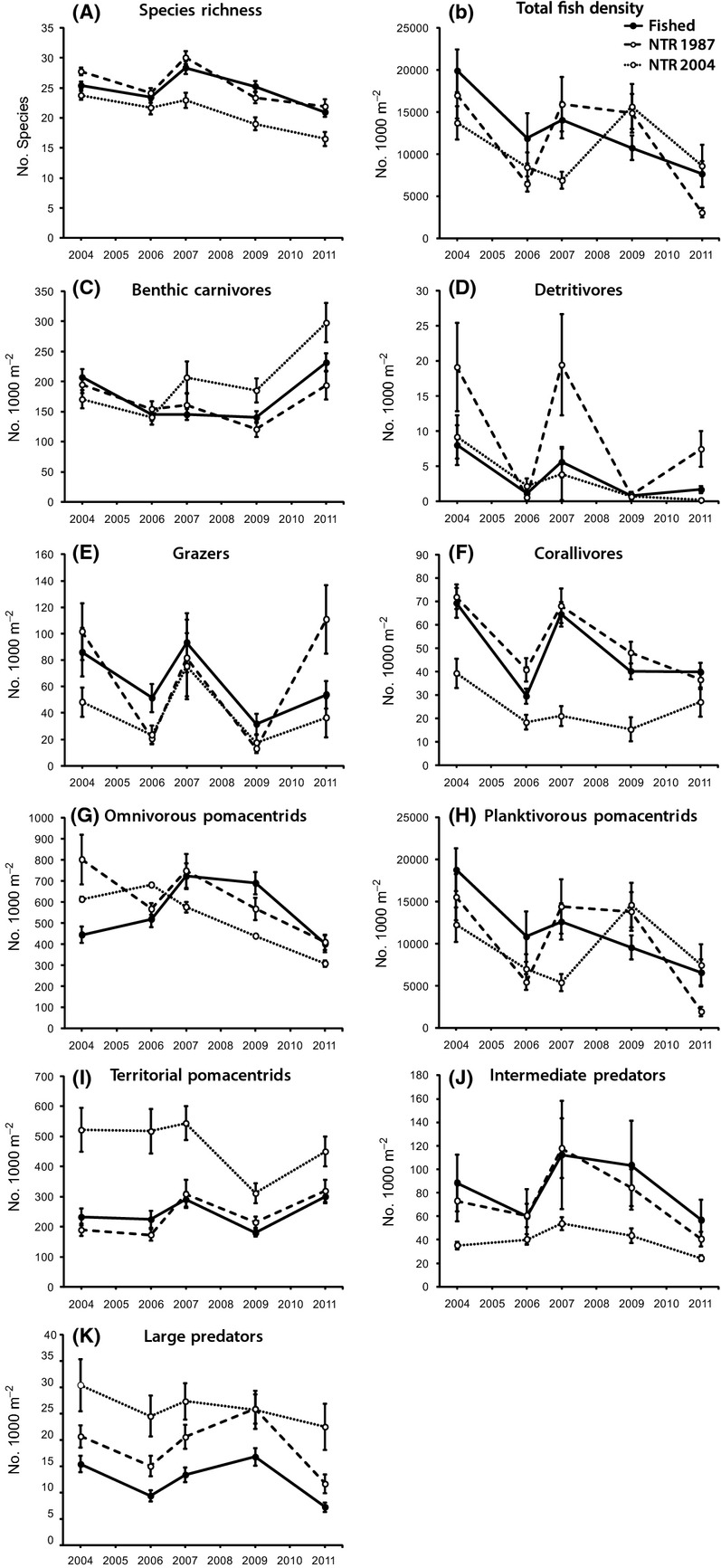
Temporal dynamics in the mean density (individuals 1000 m^−2^) of major fish functional groups, in fished zones, old NTRs (NTR 1987) and new NTRs (NTR 2004) between 2004 and 2011. Error bars are ±1 SE of the mean.

Fish species richness, total fish density (all species pooled), and the density of all analyzed functional groups, other than the intermediate predator group, changed significantly throughout the monitoring period (Fig. 5, Table 2). Fish species richness generally declined between 2006 and 2011, with a temporary recovery in 2007 in old NTRs and fished zones followed by subsequent declines. Recovery failed to occur in new NTRs, and from 2007 until 2011 species richness remained lower in new NTRs than in old NTRs and fished zones (Fig. 5A). Total fish density declined in all zones in 2006 and 2011, and this produced an overall decline throughout the monitoring period (Fig. 5B). Significant recovery of total fish density was recorded in the old NTRs between 2006 and 2007 and in the new NTRs between 2007 and 2009 (Tukey's HSD, old NTRs: *P *=* *0.03; new NTRs: *P *=* *0.02) but not in the fished zones (Fig. 5B).

Most functional groups of fishes declined in density in 2006, recovered to varying degrees between 2007 and 2009, and then declined more dramatically in 2011 (Fig. 5). This pattern was especially pronounced (and statistically significant) for corallivores, planktivorous pomacentrids, intermediate predators, and large predators (Fig. 5, Table 2). In contrast to the majority of fish groups, the mean density of benthic carnivores (Fig. 5C), grazers (Fig. 5E), and territorial pomacentrids (Fig. 5I) increased between 2009 and 2011. Trajectories of change appeared different in new NTRs for a number of groups when compared to fished zones and old NTRs. This was true for corallivores, intermediate predators (lower densities in new NTRs throughout the monitoring period), territorial pomacentrids, and large predators (higher densities throughout the monitoring period) (Fig. 5).

The relative cover of live hard coral was found to have a significant influence on fish species richness, total fish density, and on the density of all fish functional groups other than the detritivores and the intermediate predators (Table 5).

**Table 5:** Results of generalized linear mixed-effects model testing the degree of influence of live hard coral (LHC) cover on the density of fish groups.

Dependent variable	Factor_(df)_	*F*	*P*	Dependent variable	Factor_(df)_	*F*	*P*
Fish species richness	LHC_(1,471)_	4.06	<0.05	Omnivorous pomacentrids	LHC_(1,471)_	15.62	<0.001
	Year_(4,471)_	28.87	<0.001		Year_(4,471)_	9.41	<0.0001
	LHC × Year_(4,471)_	1.49	0.20		LHC × Year_(4,471)_	2.21	0.07
Total fish density	LHC_(1,471)_	15.33	<0.001	Planktivorous pomacentrids	LHC_(1,471)_	15.48	<0.001
	Year_(4,471)_	8.48	<0.0001		Year_(4,471)_	8.05	<0.0001
	LHC × Year_(4,471)_	3.71	<0.05		LHC × Year_(4,471)_	3.78	<0.05
Benthic carnivores	LHC_(1,471)_	17.94	<0.0001	Territorial pomacentrids	LHC_(1,471)_	31.05	<0.0001
	Year_(4,471)_	19.63	<0.0001		Year_(4,471)_	7.80	<0.0001
	LHC × Year_(4,471)_	1.69	0.15		LHC × Year_(4,471)_	5.05	<0.0001
Detritivores	LHC_(1,471)_	1.29	0.26	Intermediate predators	LHC_(1,471)_	0.01	0.91
	Year_(4,471)_	9.81	<0.0001		Year_(4,471)_	2.45	<0.05
	LHC × Year_(4,471)_	1.97	0.09		LHC × Year_(4,471)_	0.27	0.89
Grazers	LHC_(1,471)_	7.49	<0.05	Large predators	LHC_(1,471)_	5.09	<0.05
	Year_(4,471)_	9.19	<0.0001		Year_(4,471)_	18.01	<0.0001
	LHC × Year_(4,471)_	7.47	<0.0001		LHC × Year_(4,471)_	2.52	<0.05
Corallivores	LHC_(1,471)_	46.55	<0.0001	*Plectropomus* spp.	LHC_(1,471)_	7.15	<0.05
	Year_(4,471)_	25.44	<0.0001		Year_(4,471)_	17.26	<0.0001
	LHC × Year_(4,471)_	4.24	<0.05		LHC × Year_(4,471)_	2.43	<0.05

### Temporal dynamics in coral trout density: the effects of NTRs and disturbances

**Figure 6:**
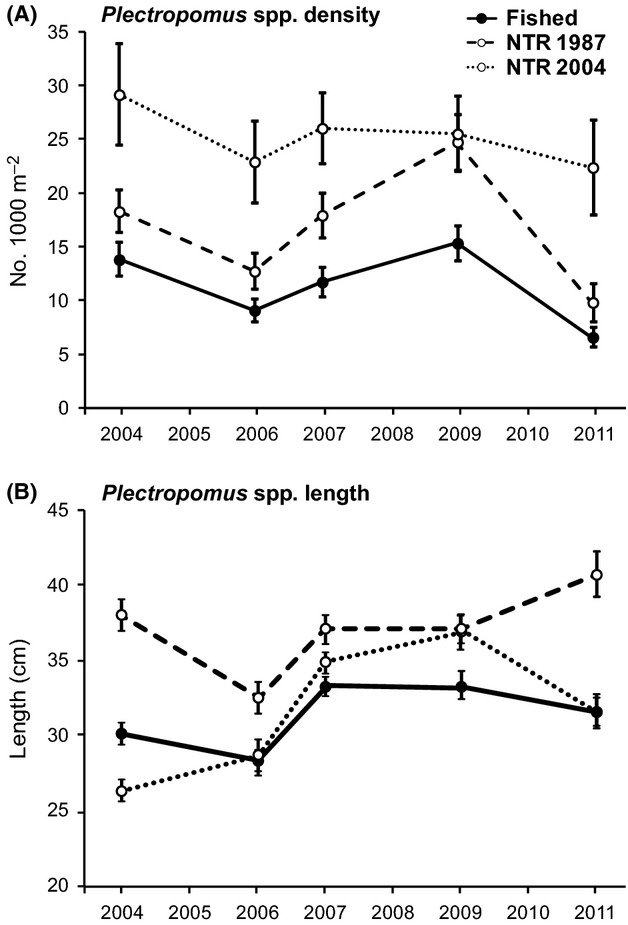
Temporal dynamics in (A) mean density (individuals 1000 m^−2^) and (B) mean length (cm-TL) of coral trout (*Plectropomu*s spp.) in fished zones, old NTRs (NTR 1987), and new NTRs (NTR 2004) between 2004 and 2011. Error bars are ±1 SE of the mean.

Two species of coral trout were recorded in the Keppel Islands during the monitoring period, *Plectropomus maculatus* and *P. leopardus*. The relative species composition was approximately 98% *P. maculatus* and 2% *P. leopardus*. The mean density of coral trout remained significantly higher in new NTRs than in fished zones in all years, and consistently higher in old NTRs than in fished zones; however, this latter difference was only significant in 2009 (Fig. 6A, Table 2). Mean coral trout density was also significantly higher in new NTRs than in old NTRs in three of the five survey years (2004, 2006, and 2011) (Tukey's HSD, *P *<* *0.05). In 2009, after 5 years of protection for the new NTRs and 3 years of post-bleaching recovery, the mean density of coral trout was essentially equal in both old and new reserves (Fig. 6A). There was no significant change in mean coral trout density within new NTRs throughout the monitoring period (Tukey's HSD, *P *>* *0.05). There was, however, a significant increase in mean coral trout density between 2006 and 2009 within old NTRs (Tukey's HSD, *P *<* *0.01) and significant declines in density following the 2011 flood plume in both old NTRs and fished zones (Tukey's HSD, *P *<* *0.001 and *P *<* *0.05, respectively). Despite these declines, in 2011, the overall magnitude of the reserve effects was maintained, with mean coral trout density ratios of 1.5:1 between old NTRs and fished zones, and 3.4:1 between new NTRs and fished zones (Fig. 6A).

The temporal dynamics of coral trout density closely tracked the trajectory of live hard coral cover (cf. Figs. 2B, 6A). As was the case for most fish functional groups, live hard coral cover was found to have a significant influence on coral trout density (Table 5).

Coral trout were larger on average in old NTRs than in fished zones and new NTRs (*F*_2,64_ = 3.66, *P *<* *0.05); however, this difference was only significant in 2004 and 2011 (Tukey's HSD, *P *<* *0.05 in both cases) (Fig. 6B). Across all survey years, the mean length of coral trout in old NTRs was 37 cm TL (±0.5 cm SE), while the mean length in both new NTRs and fished zones was 31.5 cm TL (±0.4 cm SE). Following the initial disturbance (2006), mean coral trout length declined slightly in both fished zones and old NTRs, but increased within new NTRs. Mean length continued to increase within new NTRs following the 2006 bleaching disturbance, and coral trout were significantly larger in 2007 and 2009 than they were in the pre-disturbance and pre-reserve period of 2004 (Tukey's HSD, *P *<* *0.05 in both cases). Between 2009 and 2011, mean coral trout length increased slightly in old NTRs, decreased in new NTRs, and remained relatively stable in fished zones. Within fished zones and both old and new NTRs, mean coral trout length was not significantly different between the 2004 pre-disturbance period and the 2011 post-disturbance period (Fig. 6B).

**Figure 7:**
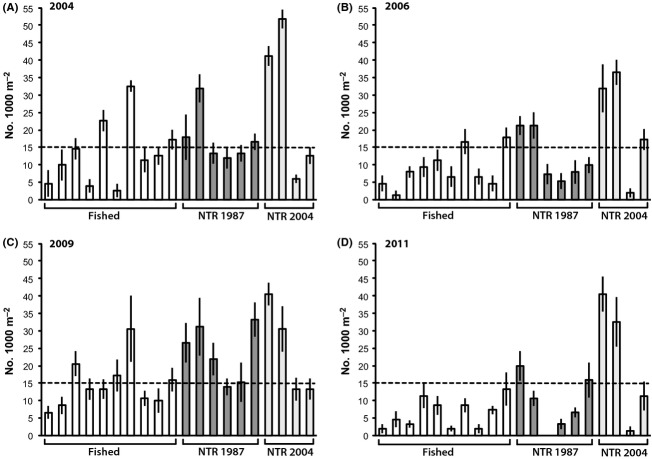
Pre-and post-disturbance refuges for coral trout (*Plectropomus* spp.) populations in the Keppel Islands. Mean density (individuals 1000 m^−2^ ± 1 SE) at each site within fished and NTR zones during the healthy reef condition (pre-disturbance) years of 2004 and 2009 and the degraded reef condition (post-disturbance) years of 2006 and 2011. The dashed line represents the defined threshold density for healthy coral trout populations in the Keppel Islands (15 fish 1000 m^−2^).

The mean coral trout density across all sites and years was 15.09 fish 1000 m^−2^ (±0.58 SE). The indicator threshold for a healthy coral trout population density was thus defined as 15 fish 1000 m^−2^. During the pre-disturbance, healthy reef condition years of 2004 and 2009, 30–40% of sites in fished zones, and 50–80% of sites in both old and new NTRs supported coral trout densities above the threshold density (Fig. 7A, C). Following the 2006 coral bleaching disturbance, 20% of fished zone sites, 33% of old NTR sites, and 75% of new NTR sites still supported healthy coral trout densities that were above the threshold (Fig. 7B). After the 2011 flood plume disturbance, however, coral trout densities had declined below the threshold at all sites within fished zones, while 33% of sites within old NTRs and 50% of sites within new NTRs had retained densities above the threshold (Fig. 7D).

## Discussion

Benthic and fish communities in the Keppel Island group were significantly impacted by both the 2006 coral bleaching and the 2011 flood plume, which resulted in relative declines in hard coral cover of 26% and 37%, respectively. The temporal dynamics of fish communities generally matched the dynamics of the benthic habitat, with live hard coral cover and associated habitat complexity being good predictors of fish density and community structure. There was no evidence that NTRs directly mitigated against the impacts of these major extrinsic disturbance events, either for benthic habitat structure or for nonexploited fish that were closely associated with the benthic habitat. The impacts of the disturbances were patchy, with some reserve sites and nonreserve sites escaping major damage, and others being severely impacted. However, despite the disturbance-induced changes to the benthos, NTRs maintained higher densities of the most important fishery species (coral trout) than fished areas. Furthermore, following the two disturbance events, the only remaining large populations of coral trout were within NTRs. Hence, the reserves that escape damage may provide critical refuges for adult fish, with larval export from these sites contributing to population recovery and long-term persistence following such massive and unpredictable disturbance events.

### Impacts of disturbance on the structure and function of benthic communities

In terms of overall hard coral cover, the benthic community recovered quickly following the 2006 bleaching event, with the mean live hard coral cover in 2009 back to the pre-disturbance levels of 2004. The recovery of hard coral cover was primarily driven by branching, plate, and digitate acroporid corals. The structure of the benthic community was significantly altered by the 2006 bleaching event, and the “recovered” community of 2009 remained significantly different to the prebleaching community. Rapid recovery from coral bleaching has been reported in other studies, including, for instance, an increase in coral cover from 24% in 1991 to 47% in 2006 in Moorea (Adjeroud et al. 2009) and from 10% in 2005 to 29% in 2009 at Ashmore Reef (Timor Sea) (Ceccarelli et al. 2011). Furthermore, Diaz-Pulido et al. (2009) documented almost complete hard coral recovery at several sites in the Keppel Islands within 6–12 months of the 2006 bleaching event. This recovery was primarily driven by vegetative recolonization of dead *Acropora* spp. branches by remnant live coral tissue and subsequent successful competition by corals over the locally prolific macroalgae, *Lobophora variegata* (Diaz-Pulido et al. 2009). The findings reported here and by several previous studies reinforce the notion that coral communities in the Keppel Islands have a high capacity to recover from disturbance. Other studies have shown that recovery of corals can be spatially and temporally variable, with coral cover recovering very slowly (Smith et al. 2008), or not at all in some cases (Bruno and Selig 2007), as seen at sites within the new NTRs in this study.

The two disturbance events documented in the present study differed in their nature and in the scale of impact on the benthic communities. The 2006 bleaching event shifted benthic communities from a dominance of live hard corals of various growth forms to dominance by dead hard corals and macroalgae. Pre-disturbance (2004) communities on the study reefs were dominated by branching and digitate acroporid species, which are particularly susceptible to bleaching (Marshall and Baird 2000; Carpenter et al. 2008). It has been demonstrated that more diverse coral communities may be more resistant to post-bleaching mortality (Furby et al. 2013). The 2011 flood plume resulted in higher overall, and morphologically more indiscriminate, mortality of hard corals than the bleaching event. Similar degradation across the entire coral community was recorded following a severe flood event in 1991 (van Woesik et al. 1995). While the bases and shaded portions of coral colonies can be protected from bleaching (van Oppen et al. 2009), freshwater penetration throughout the colonies is likely to have caused more complete colony mortality during the flood. The post-disturbance benthic community in 2011 was overwhelmingly dominated by dead branching corals, as macroalgae also suffered high mortality during the flood plume. The decline of macroalgae due to freshwater flooding has not been widely documented, but their demise may potentially facilitate faster recovery of corals through recruitment (Wilson et al. 2012). The two post-disturbance communities in 2006 and 2011 were therefore distinctly different, probably due to a combination of different starting points (pre-disturbance community-level differences in 2004 and 2009), and differences in the nature, severity, and extent of the two climatic disturbances.

### Responses of reef fishes to benthic habitat disturbance

Despite the significant recovery of live hard coral between 2006 and 2009, the overall composition of the fish functional groups had failed to return to its pre-disturbance state by 2009. The recovery of the coral community may have at least partially decelerated the decline of the fish community; however, following the 2011 disturbance, the fish community continued toward a relatively depauperate state with a proportional dominance of territorial pomacentrids and benthic carnivores (predominantly small-bodied species of Labridae). The results presented here are consistent with the findings of previous studies where declines in live coral cover and habitat complexity have been followed by declines in fish density and diversity, including within NTRs (Jones et al. 2004; Graham et al. 2006; Pratchett et al. 2011). Loss of live hard coral and shifts in the structure of coral communities will almost universally have flow-on effects for fish communities (Wilson et al. 2006; Coker et al. 2012). Most reef fishes have specific dietary and shelter preferences, and many are reliant at least to some extent on live hard coral and reef structural complexity (Pratchett et al. 2006, 2011; Verweij et al. 2006; Wilson et al. 2009; Kerry and Bellwood 2012). Thus, it was not surprising that we observed significant reductions in the density of most fish species, particularly coral-feeding butterflyfishes (Chaetodontidae) and omnivorous and planktivorous damselfishes (Pomacentridae), in response to reductions in live hard coral cover and habitat complexity. It was surprising, however, that coral trout displayed such a strong response to the loss of live hard coral, as large species at higher trophic levels have generally been found to be less affected by fine-scale changes in their habitat (Ruppert et al. 2013). Changes in hard coral cover strongly affected the abundance of prey fish species, and this may have been one of the key mechanisms driving the observed changes in the abundance of piscivores such as coral trout (Wilson et al. 2008). Furthermore, juvenile coral trout have been shown to be closely associated with patchy live coral habitats in the Keppel Islands (Wen et al. 2013a). It is probable that the observed coral declines may also have impacted the rates of post-settlement mortality, particularly in sites that contain important coral trout settlement and nursery habitats (Wen et al. 2013b).

The fate of reef fishes following habitat loss may strongly influence the rate of recovery of populations after disturbances (Bellwood et al. 2012). However, it remains unclear whether fish move or die as a result of habitat degradation, especially in complex interconnected reef systems such as the GBR. In reality, there are a range of responses to disturbance of the benthic habitat among fish species, and intuitively, it is likely that small-bodied, more site attached species will be prone to mortality following disturbance, while larger, more mobile species would have a greater capacity to relocate to healthy reef areas (Pratchett et al. 2011). Reefs with recovering benthos may become repopulated more rapidly if fish have simply moved and can return once conditions have improved. However, for fish species that experience high mortality following disturbance, successful recruitment (settlement) events will be required for population recovery. In the present study, partial recovery was documented between 2006 and 2009 in the densities of several fish groups including the planktivorous pomacentrids and the intermediate and large predators (particularly *Plectropomus* spp.). In this case, it is likely that the majority of the observed recovery in damselfish populations occurred via recruitment. However, given that the density and the mean length of coral trout generally increased in both NTRs and fished zones between 2006 and 2009, it appears likely that fish had moved out of the degraded areas following the disturbances and returned to recovering areas once adequate prey and shelter became available again.

### Role of no-take marine reserves: habitat and fish community structure

The protection of reefs within NTRs did not appear to influence the response of benthic and fish communities to the disturbances, nor did NTRs affect recovery rates between the disturbance events. Both disturbances were spatially patchy and the worst affected areas included both NTR and fished areas. The geographic location, orientation, and exposure of each site to the disturbances appeared to be a much stronger determinant of the degree of damage sustained than whether or not the site was within a NTR. It is well known that NTRs cannot provide a barrier against pervasive threats such as rising ocean temperature and acute climatic disturbances, but their primary role, supported by the results of this study, is in the maintenance of the trophic balance of marine communities and thus enhancement of reef recovery (Almany et al. 2009; Graham et al. 2011). Whether reserves in relatively lightly exploited, interconnected reef systems such as the GBR can lead to ecosystem-level changes (McClanahan et al. 2007; Sandin et al. 2008; Russ and Alcala 2011) or improved resilience (Mumby et al. 2006; Mumby and Harborne 2011) is unknown.

### No-take marine reserves and the decline and recovery of coral trout populations

The mean density of the key fishery target species, coral trout (*Plectropomus* spp.), was consistently higher on reefs within NTRs than on surrounding fished reefs. It was clear that the primary post-disturbance refuges of coral trout spawning stocks were all located within the NTRs that avoided the worst of the disturbances and retained high coral cover. During the post-disturbance, degraded reef state of 2011, the density ratio of *Plectropomus* spp. inside and outside NTRs was maintained at between 1.5:1 (old NTR: Fished) and 3.4:1 (new NTR: Fished), despite the dramatic declines in coral cover and significant shifts in fish community structure within both NTRs and fished zones. Old NTRs in particular provided both fishing and disturbance refuges for large adult coral trout, and this effect was particularly strong in both 2004 when reefs were in good condition, and in 2011, when reefs were in a relatively degraded state. New NTR sites hosted higher densities of smaller coral trout, suggesting that those reefs are important nursery areas for juvenile and subadult fish (Wen et al. 2013b). A recent study has demonstrated that coral trout populations within NTRs of the Keppel Islands were contributing over half of the total juvenile recruitment to all fished and NTR reefs in the island group during 2007 and 2008 (Harrison et al. 2012). Given the high level of self-recruitment for coral trout in the Keppel Islands, it is evident that the NTRs that provided post-disturbance refuges will be important local sources of larval production and support population recovery via recruitment.

## Conclusions

Clearly, NTRs can do little to protect coral reefs and most reef fish species from the direct impacts of large-scale acute disturbances such as coral bleaching events and flood plumes. However, our study reefs had the capacity to recover rapidly from these disturbances, as long as the disturbances did not become too frequent. The findings presented here suggest that NTR networks can effectively boost the persistence and sustainability of exploited fish populations, even in highly degraded reef systems. While the abundance of coral trout declined in response to declines in hard coral and benthic habitat complexity, densities remained consistently higher in NTRs than in fished areas. Following disturbances, the NTRs that were not damaged supported the only remaining large populations of adult coral trout.

Larval dispersal studies (Harrison et al. 2012; Almany et al. 2013) suggest that these local refuges will provide critically important local sources of recruitment for coral trout population recovery in the short term and increased population persistence in the longer term. However, this study should also serve as a stark reminder that the key focus of coral reef conservation efforts needs to apply to management “outside the reef” (Peterson et al. 2008). Declining water quality and climate change-driven increases in sea surface temperatures, ocean acidity and the intensity of extreme weather events are pervasive and will ultimately erode the natural resilience of coral reef ecosystems and undermine the benefits of NTRs. Effective NTR networks remain a powerful tool to preserve ecosystems in a state that is as natural as possible and take advantage of an innate capacity for coral reef communities to recover from disturbances. However, the need to implement strategies that address declining water quality and increasing greenhouse gas emissions at regional, national, and global scales is clear.

## Supporting Information

Table S1 Fish Functional group species allocations
