# Supplementary material for: Habitat dynamics, marine reserve status, and the decline and recovery of coral reef fish communities
Source: Ecol Evol. 2014 Jan 13;4(4):337–54. doi: 10.1002/ece3.934 (PMC3936382; doi:10.1002/ece3.934)
Supplement: Table S1 — Fish Functional group species allocations [file ece30004-0337-sd1.docx]

***Supplementary material***

**Table S1:** Fish Functional group species allocations

| *Family* | *Species* | *Functional Group* |
| --- | --- | --- |
|  |  |  |
| Acanthuridae | *Acanthurus blochii* | Detritivore |
|  | *Acanthurus dussumieri* | Detritivore |
|  | *Acanthurus grammoptilus* | Detritivore |
|  | *Acanthurus lineatus* | Algal cropper |
|  | *Acanthurus nigricauda* | Detritivore |
|  | *Acanthurus nigrofuscus* | Algal cropper |
|  | *Acanthurus xanthopterus* | Detritivore |
|  | *Ctenochaetus binotatus* | Detritivore |
|  | *Ctenochaetus striatus* | Detritivore |
|  | *Naso annulatus* | Algal cropper |
|  | *Naso brevirostris* | Algal cropper |
|  | *Naso lituratus* | Algal cropper |
|  | *Naso tuberosus* | Algal cropper |
|  | *Naso unicornis* | Algal cropper |
|  | *Prionurus microlepidotus* | Algal cropper |
|  | *Zebrasoma scopas* | Algal cropper |
|  | *Zebrasoma veliferum* | Algal cropper |
|  |  |  |
| Chaetodontidae | *Chaetodon aureofasciatus* | Corallivore |
|  | *Chaetodon auriga* | Corallivore |
|  | *Chaetodon baronessa* | Corallivore |
|  | *Chaetodon citrinellus* | Corallivore |
|  | *Chaetodon flavirostris* | Corallivore |
|  | *Chaetodon lineolatus* | Corallivore |
|  | *Chaetodon lunula* | Corallivore |
|  | *Chaetodon lunulatus* | Benthic carnivore |
|  | *Chaetodon melannotus* | Benthic carnivore |
|  | *Chaetodon ornatissimus* | Corallivore |
|  | *Chaetodon plebeius* | Corallivore |
|  | *Chaetodon rafflesi* | Corallivore |
|  | *Chaetodon rainfordi* | Corallivore |
|  | *Chaetodon speculum* | Corallivore |
|  | *Chaetodon trifascialis* | Corallivore |
|  | *Chaetodon ulietensis* | Benthic carnivore |
|  | *Chaetodon vagabundus* | Corallivore |
|  | *Chelmon rostratus* | Benthic carnivore |
|  | *Coradion altivelis* | Benthic carnivore |
|  | *Coradion chrysostomus* | Benthic carnivore |
|  | *Heniochus acuminatus* | Benthic carnivore |
|  | *Heniochus monoceros* | Benthic carnivore |
|  | *Heniochus varius* | Benthic carnivore |
|  | *Parachaetodon ocellatus* | Benthic carnivore |
|  |  |  |
| Ephippidae | *Platax orbicularis*  *Platax teira* | Benthic carnivore  Benthic carnivore |
|  | *Platax pinnatus* | Benthic carnivore |
|  | *Platax teira* | Benthic carnivore |
|  |  |  |
| Haemulidae | *Diagramma pictum* | Large predator |
|  | *Plectorhinchus chaetodontoides* | Large predator |
|  | *Plectorhinchus flavomaculatus* | Large predator |
|  | *Plectorhinchus gibbosus* | Large predator |
|  | *Plectorhinchus lessonii* | Large predator |
|  | *Plectorhinchus unicolor* | Large predator |
|  |  |  |
| Kyphosidae | *Kyphosus spp.* | Algal cropper |
|  | *Microcanthus strigatus* | Benthic carnivore |
|  |  |  |
| Labridae | *Anampses geographicus*  *Anampses neoguinaicus* | Benthic carnivore  Benthic carnivore |
|  | *Bodianus axillaris*  *Bodianus loxozonus*  *Bodianus mesothorax* | Benthic carnivore  Benthic carnivore  Benthic carnivore |
|  | *Cheilinus chlorurus* | Benthic carnivore |
|  | *Cheilinus fasciatus* | Benthic carnivore |
|  | *Cheilinus trilobatus* | Benthic carnivore |
|  | *Cheilinus undulatus* | Benthic carnivore |
|  | *Choerodon anchorago* | Benthic carnivore |
|  | *Choerodon cyanodus* | Benthic carnivore |
|  | *Choerodon fasciatus* | Benthic carnivore |
|  | *Choerodon graphicus* | Benthic carnivore |
|  | *Choerodon monostigma* | Benthic carnivore |
|  | *Choerodon schoenleinii* | Benthic carnivore |
|  | *Choerodon vitta* | Benthic carnivore |
|  | *Epibulus insidiator* | Benthic carnivore |
|  | *Gomphosus varius* | Benthic carnivore |
|  | *Halichoeres melanurus* | Benthic carnivore |
|  | *Hemigymnus fasciatus* | Benthic carnivore |
|  | *Hemigymnus melapterus* | Benthic carnivore |
|  | *Labrichthys unilineatus* | Benthic carnivore |
|  | *Labroides bicolor*  *Labroides dimidiatus* | Benthic carnivore |
|  | *Labropsis australis* | Benthic carnivore |
|  | *Oxycheilinus diagramma* | Benthic carnivore |
|  | *Psuedolabrus guentheri* | Benthic carnivore |
|  | *Stethojulis bandanensis*  *Stethojulis strigiventer* | Benthic carnivore  Benthic carnivore |
|  | *Thalassoma hardwicke*  *Thalassoma jansenii*  *Thalassoma lunare*  *Thalassoma lutescens* | Benthic carnivore  Benthic carnivore  Benthic carnivore  Benthic carnivore |
|  |  |  |
| Lethrinidae | *Gymnocranius spp.* | Intermediate predator |
|  | *Lethrinus atkinsoni* | Intermediate predator |
|  | *Lethrinus laticaudis* | Intermediate predator |
|  | *Lethrinus lentjan* | Intermediate predator |
|  | *Lethrinus miniatus* | Intermediate predator |
|  | *Lethrinus nebulosus* | Intermediate predator |
|  | *Lethrinus obsoletus* | Intermediate predator |
|  | *Lethrinus ornatus* | Intermediate predator |
|  | *Monotaxis grandoculis.* | Intermediate predator |
|  |  |  |
| Lutjanidae | *Lutjanus argentimaculatus* | Large predator |
|  | *Lutjanus carponotatus* | Intermediate predator |
|  | *Lutjanus fulviflamma* | Intermediate predator |
|  | *Lutjanus fulvus* | Intermediate predator |
|  | *Lutjanus lemniscatus* | Intermediate predator |
|  | *Lutjanus lutjanus* | Intermediate predator |
|  | *Lutjanus monostigma* | Intermediate predator |
|  | *Lutjanus quinquelineatus* | Intermediate predator |
|  | *Lutjanus russelli* | Intermediate predator |
|  | *Lutjanus sebae* | Intermediate predator |
|  | *Lutjanus vitta* | Intermediate predator |
|  | *Symphorus nematophorus* | Large predator |
|  |  |  |
| Mullidae | *Parupeneus barberinus* | Benthic carnivore |
|  | *Parupeneus bifasciatus* | Benthic carnivore |
|  | *Parupeneus ciliatus* | Benthic carnivore |
|  | *Parupeneus indicus* | Benthic carnivore |
|  |  |  |
| Muraenidae | *Echidna nebulosa* | Intermediate predator |
|  | *Gymnothorax favagineus*  *Gymnothorax javanicus*  *Gymnothorax meleagris*  *Gymnothorax undulatus* | Intermediate predator  Intermediate predator  Intermediate predator  Intermediate predator |
|  | *Gymnothorax javanicus* | Intermediate predator |
|  | *Gymnothorax meleagris* | Intermediate predator |
|  |  |  |
| Nemipteridae | *Scolopsis bilineatus* | Intermediate predator |
|  | *Scolopsis margaritifer* | Intermediate predator |
|  | *Scolopsis monogramma* | Intermediate predator |
|  |  |  |
| Pomacanthidae | *Centropyge bicolor* | Benthic carnivore |
|  | *Centropyge bispinosus* | Benthic carnivore |
|  | *Centropyge nox* | Benthic carnivore |
|  | *Centropyge tibicen* | Benthic carnivore |
|  | *Centropyge vrolikii* | Benthic carnivore |
|  | *Chaetodontoplus douboulayi* | Benthic carnivore |
|  | *Chaetodontoplus meredithi* | Benthic carnivore |
|  | *Pomacanthus imperator* | Benthic carnivore |
|  | *Pomacanthus semicirculatus* | Benthic carnivore |
|  | *Pomacanthus sexstriatus* | Benthic carnivore |
|  | *Pomacanthus xanthometapon* | Benthic carnivore |
|  | *Pygoplites diacanthus* | Benthic carnivore |
|  |  |  |
| Pomacentridae | *Abudefduf bengalensis*  *Abudefduf sexfasciatus*  *Abudefduf vaigiensis*  *Abudefduf whitleyi* | Omnivorous pomacentrid  Omnivorous pomacentrid  Omnivorous pomacentrid  Omnivorous pomacentrid |
|  | *Abudefduf sexfasciatus* | Omnivorous pomacentrid |
|  | *Abudefduf vaigiensis* | Omnivorous pomacentrid |
|  | *Abudefduf whitleyi* | Omnivorous pomacentrid |
|  | *Acanthochromis polyacanthus* | Omnivorous pomacentrid |
|  | *Amblyglyphidodon aureus* | Omnivorous pomacentrid |
|  | *Amblyglyphidodon curacao* | Omnivorous pomacentrid |
|  | *Amblyglyphidodon leucogaster* | Omnivorous pomacentrid |
|  | *Amphiprion akindynos*  *Amphiprion chrysopterus*  *Amphiprion clarkia*  *Amphiprion melanopus*  *Amphiprion perideraion* | Omnivorous pomacentrid  Omnivorous pomacentrid  Omnivorous pomacentrid  Omnivorous pomacentrid  Omnivorous pomacentrid |
|  | *Chromis amboinensis* | Planktivorous pomacentrid |
|  | *Chromis atripectoralis* | Planktivorous pomacentrid |
|  | *Chromis atripes* | Planktivorous pomacentrid |
|  | *Chromis nitida* | Planktivorous pomacentrid |
|  | *Chromis retrofasciatus* | Planktivorous pomacentrid |
|  | *Chromis ternatensis* | Planktivorous pomacentrid |
|  | *Chromis weberi* | Planktivorous pomacentrid |
|  | *Chrysiptera rex*  *Chrysiptera rollandi* | Omnivorous pomacentrid  Omnivorous pomaceNTMRid |
|  | *Chrysiptera talboti* | Omnivorous pomacentrid |
|  | *Dascyllus aruanus*  *Dascyllus melanurus*  *Dascyllus trimaculatus*  *Dascyllus reticulatus* | Omnivorous pomacentrid  Omnivorous pomacentrid  Omnivorous pomacentrid  Omnivorous pomacentrid |
|  | *Dischistodus melanotus*  *Dischistodus perspicillatus*  *Dischistodus prosopotaenia*  *Dischistodus pseudochrysopoecilus* | Territorial pomacentrid  Territorial pomacentrid  Territorial pomacentrid  Territorial pomacentrid |
|  | *Hemiglyphidodon plagiometapon* | Territorial pomacentrid |
|  | *Neoglyphidodon melas* | Territorial pomacentrid |
|  | *Neoglyphidodon nigroris* | Territorial pomacentrid |
|  | *Plectroglyphidodon dickii* | Territorial pomacentrid |
|  | *Plectroglyphidodon lacrymatus* | Territorial pomacentrid |
|  | *Pomacentrus adelus* | Territorial pomacentrid |
|  | *Pomacentrus amboinensis* | Omnivorous pomacentrid |
|  | *Pomacentrus australis* | Omnivorous pomacentrid |
|  | *Pomacentrus bankanensis* | Territorial pomacentrid |
|  | *Pomacentrus brachialis* | Omnivorous pomacentrid |
|  | *Pomacentrus chrysurus* | Territorial pomacentrid |
|  | *Pomacentrus coelestis* | Omnivorous pomacentrid |
|  | *Pomacentrus lepidogenis* | Planktivorous pomacentrid |
|  | *Pomacentrus moluccensis* | Omnivorous pomacentrid |
|  | *Pomacentrus nagasakiensis* | Omnivorous pomacentrid |
|  | *Pomacentrus vaiuli* | Territorial pomacentrid |
|  | *Pomacentrus wardi* | Territorial pomacentrid |
|  | *Stegastes apicalis* | Territorial pomacentrid |
|  | *Stegastes fasciolatus* | Territorial pomacentrid |
|  |  |  |
| Scaridae | *Bolbometapon muricatum* | Excavating scarid |
|  | *Cetoscarus bicolor* | Excavating scarid |
|  | *Chlorurus bleekeri* | Excavating scarid |
|  | *Chlorurus microrhinus* | Excavating scarid |
|  | *Chlorurus sordidus* | Excavating scarid |
|  | *Hipposcarus longiceps* | Excavating scarid |
|  | *Scarus altipinnis* | Scraping scarid |
|  | *Scarus chamaeleon* | Scraping scarid |
|  | *Scarus dimidiatus* | Scraping scarid |
|  | *Scarus flavipectoralis* | Scraping scarid |
|  | *Scarus frenatus* | Scraping scarid |
|  | *Scarus ghobban* | Scraping scarid |
|  | *Scarus globiceps* | Scraping scarid |
|  | *Scarus niger* | Scraping scarid |
|  | *Scarus psittacus* | Scraping scarid |
|  | *Scarus rivulatus* | Scraping scarid |
|  | *Scarus rubroviolaceus* | Scraping scarid |
|  | *Scarus schlegeli* | Scraping scarid |
|  | *Scarus spinus* | Scraping scarid |
|  | *Scarus tricolor* | Scraping scarid |
|  |  |  |
| Serranidae | *Aethaloperca rogga* | Intermediate predator |
|  | *Anyperodon leucogrammicus* | Large predator |
|  | *Cephalopholis boenak* | Intermediate predator |
|  | *Cephalopholis cyanostigma* | Intermediate predator |
|  | *Cephalopholis microprion* | Intermediate predator |
|  | *Cromileptes altivelis* | Large predator |
|  | *Diploprion bifasciatus* | Intermediate predator |
|  | *Epinephelus caerulopunctatus* | Large predator |
|  | *Epinephelus fasciatus* | Intermediate predator |
|  | *Epinephelus fuscoguttatus* | Large predator |
|  | *Epinephelus lanceolatus* | Large predator |
|  | *Epinephelus merra* | Intermediate predator |
|  | *Epinephelus ongus* | Intermediate predator |
|  | *Epinephelus quoyanus* | Intermediate predator |
|  | *Plectropomus laevis* | Large predator |
|  | *Plectropomus leopardus* | Large predator |
|  | *Plectropomus maculatus* | Large predator |
|  |  |  |
| Siganidae | *Siganus argenteus* | Algal cropper |
|  | *Siganus corallinus* | Algal cropper |
|  | *Siganus doliatus* | Algal cropper |
|  | *Siganus fuscescens* | Algal cropper |
|  | *Siganus javus* | Algal cropper |
|  | *Siganus lineatus* | Algal cropper |
|  | *Siganus puellus* | Algal cropper |
|  | *Siganus punctatus* | Algal cropper |
|  | *Siganus spinus* | Algal cropper |
|  | *Siganus vulpinus* | Algal cropper |
|  |  |  |
| Zanclidae | *Zanclus cornutus* | Benthic carnivore |
